# Somatic and germline mutation in *GRIM-19*, a dual function gene involved in mitochondrial metabolism and cell death, is linked to mitochondrion-rich (Hürthle cell) tumours of the thyroid

**DOI:** 10.1038/sj.bjc.6602547

**Published:** 2005-04-19

**Authors:** V Máximo, T Botelho, J Capela, P Soares, J Lima, A Taveira, T Amaro, A P Barbosa, A Preto, H R Harach, D Williams, M Sobrinho-Simões

**Affiliations:** 1 IPATIMUP-Institute of Molecular Pathology and Immunology of the University of Porto, Rua Dr Roberto Frias s/n, 4200-465 Porto, Portugal; 2 Department of Surgery, Hospital São João, Porto, Portugal; 3 Department of Pathology, Medical Faculty of Porto, Porto, Portugal; 4 Department of Pathology, Portuguese Oncology Institute, Porto, Portugal; 5 Department of Endocrinology, Portuguese Oncology Institute, Porto, Portugal; 6 Pathology Service, ‘Dr A Onãtivia’ Hospital, Salta, Argentina; 7 Strangeways Research Laboratory, University of Cambridge, Cambridge, UK; 8 Department of Pathology, Hospital São João, Porto, Portugal

**Keywords:** thyroid cancer, familial thyroid carcinoma, Hürthle cell tumours, *GRIM-19*, mitochondrial proteins

## Abstract

Oxyphil or Hürthle cell tumours of the thyroid are characterised by their consistent excessive number of mitochondria. A recently discovered gene, *GRIM-19* has been found to fulfil two roles within the cell: as a member of the interferon-*β* and retinoic acid-induced pathway of cell death, and as part of the mitochondrial Complex I assembly. In addition, a gene predisposing to thyroid tumours with cell oxyphilia (*TCO*) has been mapped to chromosome 19p13.2 in one family. A cluster of genes involved in mitochondrial metabolism occurs in this region; one of these is *GRIM-19.* We have searched for *GRIM-19* mutations in a series of 52 thyroid tumours. Somatic missense mutations in *GRIM-19* were detected in three of 20 sporadic Hürthle cell carcinomas. A germline mutation was detected in a Hürthle cell papillary carcinoma arising in a thyroid with multiple Hürthle cell nodules. No mutations were detected in any of the 20 non-Hürthle cell carcinomas tested, nor in any of 96 blood donor samples. In one of the sporadic Hürthle cell papillary carcinomas positive for *GRIM-19* mutation, we have also detected a ret/PTC-1 rearrangement. No *GRIM-19* mutations were detected in any of the six cases of known familial Hürthle cell tumour tested, so that our results do not support the identification of *GRIM-19* as the *TCO* gene. The *GRIM-19* mutations we have detected are the first nuclear gene mutations specific to Hürthle cell tumours to be reported to date; we propose that such mutations can be involved in the genesis of sporadic or familial Hürthle cell tumours through the dual function of GRIM-19 in mitochondrial metabolism and cell death.

Oxyphil or Hürthle cell thyroid tumours constitute an unusual form of neoplasm composed of cells with a great increase in mitochondrial number, corresponding morphologically to their voluminous, granular, eosinophilic cytoplasm (Hamperl, 1962; Nesland *et al*, 1985; Sobrinho-Simões *et al*, 1985; Rosai *et al*, 1992; Sobrinho-Simões, 1995). The excessive numbers of mitochondria are found in all tumour cells, both in adenomas and in carcinomas. Large deletions of mitochondrial DNA (mtDNA), as well as somatic mutations of some mitochondrial genes are the hallmark of Hürthle cell tumours (Máximo and Sobrinho-Simões, 2000a; Máximo *et al*, 2002). Hürthle cell tumours are multiple in a proportion of cases; the multiple tumours more often occur in younger patients than the solitary tumours, suggesting a germline defect (Katoh *et al*, 1998). Dominant inheritance has only rarely been reported; a study of one pedigree with familial Hürthle cell tumours found a linkage to chromosome 19p13.2, but the identity and function of this *TCO* gene remains unknown (Canzian *et al*, 1998).

Recently, a novel gene, *GRIM-19*, has been identified. It is one of several genes associated with retinoid-interferon-induced mortality (*GRIM*) that have been reported in the literature (Hofmann *et al*, 1998). *GRIM-19* is a cell death regulatory gene that promotes apoptosis, is a negative regulator of cell growth and is also involved in mitochondrial metabolism (Angell *et al*, 2000; Lufei *et al*, 2003), it has been mapped to human chromosome 19p13.2 (Chidambaram *et al*, 2000). *GRIM-19* is the human homologue of the bovine subunit of the mitochondrial NADH:ubiquinone oxireductase complex (Complex I) of the mitochondrial respiratory chain (MRC) (Fearnley *et al*, 2001). Like cytochrome *c*, which has a role in the induction of the cell's apoptotic programme and in the MRC (Liu *et al*, 1996), and endonuclease G, which is released from mitochondria during apoptosis and subsequently translocated to the nucleus (Li *et al*, 2001), GRIM-19 has been found to fulfil two roles within the cell: as a member of the interferon-*β* and retinoic acid-induced pathway of cell death (Angell *et al*, 2000), and as part of the mitochondrial Complex I assembly (Fearnley *et al*, 2001). These two seemingly disparate functions may be linked, through the involvement of mitochondria in apoptotic cell death.

The functional importance of GRIM-19 has been recently highlighted by knockout experiments. Huang *et al* (2004) generated mice deficient in *GRIM-19* by gene targeting, and showed that homologous deletion of *GRIM-19* causes embryonic lethality at embryonic day 9.5. Interestingly, *GRIM-19*^−/−^ blastocysts display abnormal mitochondrial structure, morphology and cellular distribution (Huang *et al*, 2004).

The consistent linkage of increased mitochondrial number and increased cell growth that characterises Hürthle cell tumours suggests that one gene could be involved in both features. The dual role of GRIM-19 in apoptosis and mitochondrial biogenesis makes it a good candidate for being a gene involved in Hürthle cell tumorigenesis. Its localisation to the same region as the *TCO* gene (chromosome 19p13.2) also suggested that it could be involved in the aetiopathogenesis of familial Hürthle cell tumours. We have therefore searched for *GRIM-19* mutations in Hürthle cell tumours, and in controls.

Lufei *et al* (2003) and Zhang *et al* (2003) have demonstrated that the major role of GRIM-19 in control of cell growth is exerted through STAT3, a transcription factor known to be inhibited by GRIM-19 binding (Lufei *et al*, 2003; Zhang *et al*, 2003). Signal transducers and activators of transcription (STATs) are a family of latent cytoplasmic transcription factors that are activated after recruitment by the cytokine membrane receptors and subsequent phosphorylation. STAT proteins form homo- or heterodimers by reciprocal interactions between SH2 domains and phosphorylated tyrosine residues, translocate to the nucleus, bind to DNA and regulate their target gene expression (Darnell *et al*, 1994). It was suggested that activation of STAT3 may contribute to the loss of cell growth control, therefore leading to carcinogenesis (Grandis *et al*, 2000), by inducing elevated expression of genes involved in controlling fundamental cellular processes such as *cyclin D1* (Fukada *et al*, 1998), *c-Myc* (Bromberg *et al*, 1999), *p21*^*WAF1/CIP1*^ (Chin *et al*, 1996), as well as *VEGF* (Niu *et al*, 2002) and *ICAM1* (Caldenhoven *et al*, 1996; Cantwell *et al*, 1998; Hwang *et al*, 2003). Owing to the difficulty in studying the functional impairment of mutated *GRIM*-19 directly in formalin-fixed tissue, we have investigated the levels of expression of *ICAM1* in our series in an attempt to provide indirect evidence of the putative loss of function of GRIM-19.

Since ret/PTC rearrangements and *B-RAF* mutations are particularly prevalent in papillary carcinomas regardless of whether or not they have Hürthle cell features (Grieco *et al*, 1990; Soares *et al*, 1998, 2003; Sugg *et al*, 1998; Cohen *et al*, 2003; Kimura *et al*, 2003), we have also searched for the aforementioned genetic alterations in Hürthle cell carcinomas with *GRIM-19* mutations.

## MATERIALS AND METHODS

### Material

We studied 26 sporadic Hürthle cell tumours (five adenomas, 11 cases of the Hürthle cell variant of follicular carcinoma and 10 cases of the Hürthle cell variant of papillary carcinoma, all showing papillary carcinoma nuclear features), six carcinomas from two families with Hürthle cell tumours, 20 cases of non-Hürthle cell carcinomas (10 follicular carcinomas and 10 papillary carcinomas) (DeLellis *et al*, 2004) and 96 blood donor samples. In one of the families (four cases) there was known linkage to chromosome 19p13.2. The study was approved by the Ethical Committees of all Institutions involved and informed consent was obtained from all individuals studied.

### DNA extraction

DNA was extracted from microdissected frozen and/or paraffin-embedded tissue pairs (tumour and adjacent ‘normal’ thyroid) using the NucleoSpin® Tissue Kit (Macherey-Nagel, Düren, Germany). DNA from blood of some patients and from blood donor samples was also extracted using the same procedure.

### Screening of *GRIM-19* mutations

We searched for mutations in all five exons of *GRIM-19* including intronic boundaries (primer sequences shown in Table 1) using PCR/Automated sequencing. All PCR amplifications were performed in a 25 *μ*l volume containing 200 *μ*M of each dNTP, 12.5 pmol of each of the forward and reverse primers, 50 mM KCl, 10 mM Tris-HCl (pH 9.0), 1.5 mM MgCl_2_ and 1 U of *Taq* DNA polymerase (Amersham Pharmacia Biotech). Cycling conditions were a single predenaturation step at 94°C for 5 min followed by 35 cycles of denaturation at 94°C for 20 s, annealing at 60°C for 20 s and elongation at 72°C for 30 s, and a final incubation at 72°C for 5 min. PCR products were separated by electrophoresis on 2% agarose gels and purified using the NucleoSpin® Extract Kit (Macherey-Nagel, Düren, Germany). Sequencing analysis was then carried out on purified products using the ABI Prism BigDye™ Terminator Ready Reaction Kit (Perkin-Elmer, Foster City, CA, USA) and an ABI prism 377 DNA sequencer (Perkin-Elmer). Both strands were screened using the original primers. All altered samples were subjected to an additional complete analysis.

### Loss of heterozygosity analysis

Two markers (D19S916 and D19S413) located in the *TCO* (19p13.2) region were used for the loss of heterozygosity (LOH) studies with [^32^P]dCTP (Amersham, UK) radioactive PCR amplification. Cycling conditions were a single predenaturation step at 94°C for 5 min followed by 35 cycles of denaturation at 94°C for 20 s, annealing at 58°C for 20 s and elongation at 72°C for 30 s, and a final incubation at 72°C for 5 min. Amplicons were separated on 6% polyacrylamide denaturing gels, and exposed to X-ray film at room temperature. Loss of heterozygosity was determined by comparing the intensity of the alleles in heterozygosity samples of matched tumour and normal DNA. Loss of heterozygosity analysis was performed in all cases of sporadic (*n*=26) Hürthle cell tumours.

### Evaluation of the expression of *ICAM1*

To obtain an indirect evaluation of the functional activity of GRIM-19, we calculated the relative expression of *ICAM1* (tumour tissue *vs* normal adjacent tissue) in 26 Hürthle cell tumours with and without *GRIM-19* mutations. *ICAM1* is known to be upregulated by STAT3, a transcription factor whose function is inhibited by GRIM-19 protein (Lufei *et al*, 2003; Zhang *et al*, 2003). *ICAM1* expression was performed as follows. For each sample 1.0 *μ*g of RNA was reverse transcribed in a reaction volume of 20 *μ*l in the presence of 4 mM dNTP, 1.0 U *μ*l^−1^ RNase inhibitor, 2.5 *μ*M random primer P (dN)_6_ and 10 U *μ*l^−1^ M-MLV reverse transcriptase. Reverse-transcribed cDNAs (0.25 *μ*g) from normal and neoplastic tissues were used to coamplify the housekeeping gene – *glyceraldehyde-3-phosphate dehydrogenase* (*GAPDH*) (primers GAPDHF – tgt cag tgg tgg gac ctg acc t – and GAPDHR – cac cct gtt gct gta gcc aaa tt; amplicon: 254 bp), and the differentially expressed gene – *ICAM1* gene (primers ICAM1F – caa ccg gaa ggt gta tga act ga – and ICAM1R – tgg cag cgt agg gta agg ttc tt; amplicon: 186 bp). Cycling conditions were a single predenaturation step at 94 °C for 5 min followed by 24 cycles of denaturation at 94 °C for 20 s, annealing at 58 °C for 20 s and elongation at 72 °C for 20 s, and a final incubation at 72 °C for 5 min. The PCR products were separated in an agarose gel (2%), and stained with ethidium bromide. The intensity of the fluorescence was automatically measured and integrated using the *Multi-Analyst – version 1.1 –* software in the BIO RAD Gel DOC 1000 (*BIO RAD*, CA, USA). All the quantitations were performed in triplicate.

### Screening of ret/PTC rearrangements

RNA extracted from paraffin-embedded tumour tissue of patients who presented *GRIM-19* mutations was used for detecting ret/PTC rearrangements following the procedures previously described (Finn *et al*, 2003).

### Screening of *B-RAF* mutations

To screen for *B-RAF* mutations, we analysed DNA extracted from paraffin-embedded tumour tissue and adjacent thyroid tissue of patients who presented GRIM-19 mutations following the procedures previously described (Davies *et al*, 2002; Soares *et al*, 2003).

### Statistical analysis

The statistical analysis of the results was performed using *χ*^2^ test with the Yates correction, Fisher's exact test and Student's *t*-test. A *P*-value of <0.05 was considered statistically significant.

## RESULTS

### Screening of *GRIM-19* mutations

**Figure 1:**
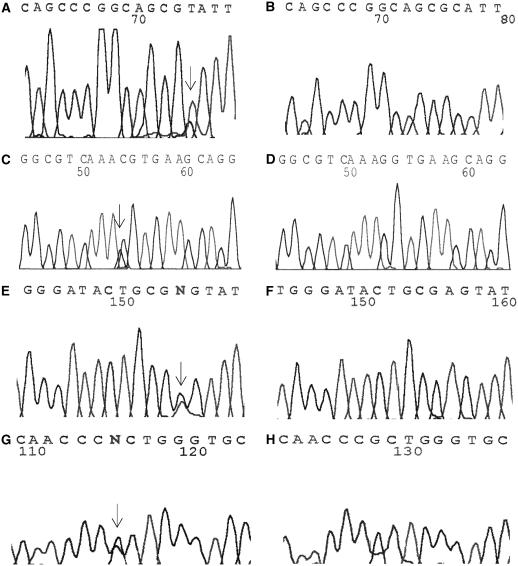
Electropherograms showing *GRIM-19* mutations in case 4 (**A** – mutated sequence, **B** – wild-type sequence), case 5 (**C** – mutated sequence, **D** – wild-type sequence), case 6 (**E** – mutated sequence, **F** – wild-type sequence) and case 7 (**G** – mutated sequence, **H** – wild-type sequence). The mutated nucleotides are indicated by the arrow. For details see Table 2.

The results of GRIM-19 analysis are summarized in Table 2 and Figure 1. Sequence determination of the five exons of *GRIM-19* in the 26 apparently sporadic Hürthle cell tumours disclosed the existence of four mutations: a C → T substitution at nucleotide 77 (exon 1) resulting in an alanine-to-valine change at residue 26 in patient 4 (Hürthle cell variant of follicular carcinoma); a G → C substitution at nucleotide 264 resulting in a lysine-to-asparagine change at residue 88 (exon 1) in patient 5 (Hürthle cell variant of papillary carcinoma with Warthin's like features) (Figure 1); an A → G substitution at nucleotide 247 resulting in a serine-to-glycine change at residue 83 (exon 1) in patient 6 (Hürthle cell variant of papillary carcinoma); and a G → C substitution at nucleotide 593 resulting in a arginine-to-proline change at residue 198 (exon 5) in patient 7 (Hürthle cell variant of papillary carcinoma). All mutations detected were heterozygous.

In patient 5, the thyroid tissue apart from the papillary carcinoma was almost totally occupied by several nodules composed of Hürthle cells; in all samples tested, including the carcinoma, one benign nodule and the internodular normal tissue, we detected the same mutation (a G → C substitution at nucleotide 264 resulting in a lysine-to-asparagine change at residue 88). The same mutation was also detected in the peripheral blood of the patient thus confirming its germline nature. The germline nature of the mutation was confirmed by its detection in the peripheral blood of one son, aged 41 years, with no clinical or ecographic signs of thyroid disease. The mutation was not detected in the other son nor in the daughter of the patient. No mutations were detected in the adjacent normal thyroid parenchyma of the other three cases with a *GRIM-19* mutation (patients 4, 6 and 7), nor in any of the 42 other normal thyroid samples.

We did not detect mutations in the *GRIM-19* gene in any of the six cases of known familial Hürthle cell tumours, in any of the 20 cases of non-Hürthle follicular and papillary carcinomas, nor in any of the 96 blood donor samples. The frequency of *GRIM-19* somatic mutations in the cases of Hürthle cell variant of follicular carcinoma (one out of 11; 9.1%) is not statistically different from the frequency of *GRIM-19* somatic mutations in cases of Hürthle cell variant of papillary carcinoma (two out of 10; 20.0%) (*P*=0.476); the same holds true if the case presenting the *GRIM-19* germline mutation is included (three out of 10; 30.0%) (*P*=0.223). None of the previously described polymorphisms (National Center for Biotechnology Information (http://www.ncbi.nlm.nih.gov)) occurring at exonic regions were detected in these cases.

**Figure 2:**
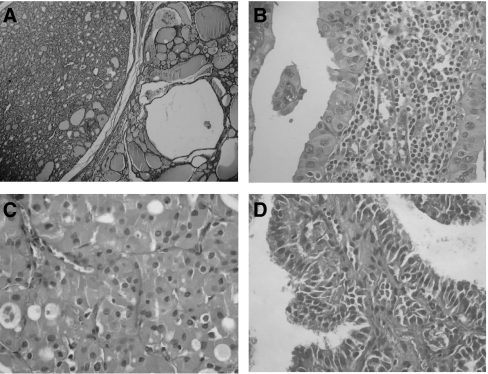
Histology of *GRIM-19* mutated tumours. **A** and **B** – Hürthle cell lesions from case 5 presenting GRIM-19 germline mutations (**A** – Hürthle cell adenoma; **B** – Hürthle cell variant of papillary carcinoma). (**C**) Hürthle cell variant of follicular carcinoma (case 4) displaying somatic GRIM-19 mutation. (**D**) Hürthle cell variant of papillary carcinoma (case 7) presenting a somatic *GRIM-19* mutation and a ret/PTC-1 rearrangement.

The presence of *GRIM-19* mutations was not significantly associated with the age and/or gender of the patients (data not shown) nor with the histotype of the lesions other than the oxyphilia (Figure 2), including the presence or absence of lymphocytic thyroiditis. No significant association was also found between the presence of *GRIM-19* mutations and the mtDNA somatic mutations detected in a previous study (Máximo *et al*, 2002) (data not shown).

### Loss of heterozygosity

The 26 cases in which LOH analysis was performed were informative for at least one of the two markers. We did not find LOH in any of the four Hürthle cell tumours presenting *GRIM-19* mutations, nor in any of the other 22 sporadic Hürthle cell tumours.

### Expression of *ICAM1*

The results are summarized in Table 3. The four tumours with mutations had significantly (*P*<0.001) higher levels of *ICAM1* expression in tumoural tissue *vs* normal tissue (range of the ratio between the expressions of *ICAM1* in tumoural tissues *vs* normal tissue: 3.2–5.0; mean±standard deviation=3.9±0.8) (Table 3) than the 22 cases without *GRIM*-19 mutation (range: 0.9–2.4; mean±standard deviation=1.4±0.4). Only two cases without *GRIM*-19 mutation (case 12, HCPC and case 15, HCPC) showed a level of expression in tumoural tissue higher than 2 (2.3 and 2.4, respectively).

### Screening for RET/PTC rearrangements and *B-RAF* mutations

The data concerning the screening for RET/PTC rearrangements and *B-RAF* mutations in cases displaying *GRIM-19* mutation are summarized in Table 4 (case 6 – not studied for technical limitations). Ret/PTC-1 chimeric transcripts were detected in one case (Case 7); no ret/PTC-3 chimeric transcripts were detected in any case. No mutations in exons 11 and 15 of *B-RAF* were detected in any of the three cases from which material was available.

## DISCUSSION

**Figure 3:**
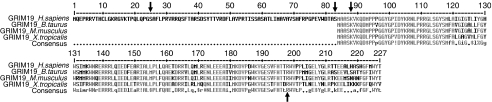
Alignment of the GRIM-19 protein sequence in four species (*Homo sapiens*, *Bos taurus*, *Mus musculus* and *Xenopus tropicalis*; accession number: NP_057049, NP_788845, NP_075801 and NP_988900, respectively). Arrows indicate the positions of the amino acids mutated in our series of Hürthle cell tumours (see Table 2).

The *GRIM-19* mutations we have described are the first nuclear gene mutations specific to Hürthle cell tumours to be reported to date. The detection of *GRIM-19* mutations in four of 26 apparently sporadic Hürthle cell tumours, and their absence in 20 non-Hürthle cell tumours and 96 blood donors support the hypothesis that alterations of *GRIM-19* are involved in the etiopathogenesis of these tumours. Further support comes from a search for germline mutations. While these were not found in the two known families studied, one germline mutation was found in the 142 samples tested (46 normal thyroids and 96 blood donor samples), and this occurred in the only apparently sporadic tumour with multiple Hürthle cell lesions. None of the alterations detected were previously described as polymorphisms, suggesting that might affect the function of the protein, or that they are rare polymorphisms. When we compare the amino-acid sequence of the protein GRIM-19 of four different species (*Homo sapiens*, *Bos taurus*, *Mus musculus* and *Xenopus tropicalis*), two of the mutations are located in phylogenetically conserved positions (K88N, case 5 and R198P, case 7) (Figure 3), suggesting that they might directly affect the function of the protein. The remaining mutations (A26V, case 4 and S83G, case 6) are located in a region of human GRIM-19 protein that has no correspondence to the GRIM-19 of other species; human GRIM-19 is the largest of the four.

In contrast to the absence of other documented nuclear genetic alterations, several mtDNA alterations (including large deletions and somatic mutations) have been described in sporadic Hürthle cell tumours (Tallini *et al*, 1994; Máximo and Sobrinho-Simões, 2000b; Máximo *et al*, 2002). Curiously, most of the somatic mutations of the mtDNA on record involve genes that, like *GRIM-19*, code for components of Complex I of MRC (Yeh *et al*, 2000; Máximo *et al*, 2002). These genes are crucial for the oxidative phosphorylation process and consequently for the cell's energy production, and it has been suggested that mutations in genes directly or indirectly affecting mitochondrial function could be the cause of the increase in mitochondrial number in Hürthle cell tumours (Katoh *et al*, 1998). It is known that a defect in the energy production machinery of the cell can lead to a secondary increase in the number of mitochondria, through a feedback mechanism (Attardi *et al*, 1995). Loss of function of *GRIM-19* can therefore explain the mitochondrial excess typical of Hürthle cell tumours.

Mutations in *GRIM-19* as well as affecting mitochondrial number could also influence cell growth and apoptosis. This role of *GRIM-19* was demonstrated using *in vitro* models by Angell *et al* (2000), who showed that overexpression of *GRIM-19* enhanced cell death in MCF-7 cells, whereas its downregulation, as well as its deletion, provided growth advantage and decreased cell death (Angell *et al*, 2000). Huang *et al* (2004) confirmed that GRIM-19 protein cellular localisation in various cell types is primarily mitochondrial. Furthermore, GRIM-19 is detected in the native form of mitochondrial complex I and the elimination of *GRIM-19* destroys the assembly and electron transfer activity of Complex I and also influences the other complexes in the mitochondrial respiratory chain (Huang *et al*, 2004).

In this study, *GRIM-19* mutations were found in 16% of Hürthle cell tumours. However *GRIM-19* is one of a number of genes that code for proteins involved in mitochondrial electron transport. Clusters of these genes occur at 19p13.2 (http://www.ncbi.nlm.nih.go v) and at 17p13 (Farrand *et al*, 2002), both regions of the genome linked to Hürthle cell tumours. It is therefore likely that mutations in other genes with similar functions are involved in the causation of Hürthle cell tumours of the thyroid, and they should also be considered candidate genes for mitochondrial-rich tumours of other sites.

The major role of GRIM-19 in control of cell growth is exerted through STAT3, a transcription factor known to be inhibited by GRIM-19 binding (Lufei *et al*, 2003; Zhang *et al*, 2003). We have shown that the cases with *GRIM*-19 mutations display increased expression of the STAT3 regulated gene *ICAM1*; this finding fits with a loss of function of GRIM-19. Only two of the 22 cases without mutation in *GRIM-19* gene showed a slightly elevated expression of the *ICAM1* gene: the levels in these two cases were considerably lower (*P*=0.07) than those detected in four cases with mutations and may reflect alterations in pathways other than STAT3 pathway.

The failure to detect LOH with the limited range of markers used does not exclude a tumorigenic role for GRIM19 mutations. Angell *et al* (2000) showed that downregulation of *GRIM-19* was able to provide growth advantage, and cooperating mutations in one of the many other nuclear or mitochondrial genes with similar functions could also be important in this setting.

Ret/PTC rearrangements were initially reported to be restricted to conventional papillary thyroid carcinomas (Grieco *et al*, 1990). Later, Cheung *et al* (2000) demonstrated that ret/PTC rearrangements could also be detected in cases of Hürthle cell variant of papillary carcinoma, thus supporting the assumption that ret/PTC is specific for the papillary phenotype (Cheung *et al*, 2000; DeLellis *et al*, 2004). Chiappetta *et al* (2002) reported the presence of the rearrangement in both benign and malignant Hürthle thyroid tumours including follicular and papillary histotypes; this finding remains to be confirmed in other series. *B-RAF* mutations have also been frequently detected in cases of Hürthle cell variant of papillary carcinoma (Trovisco *et al*, 2004), but not in other types of Hürthle cell tumour (Kimura *et al*, 2003; Soares *et al*, 2003). In order to verify if the *GRIM-19* mutation may cooperate with those genetic events in Hürthle cell tumours, we have searched for them in *GRIM-19*-positive cases. The detection of a ret/PTC-1 rearrangement in Case 7 (Hürthle cell variant of papillary carcinoma) suggests that *GRIM-19* mutation may serve as a predisposing alteration for the occurrence of tumours with cell oxyphilia. Other alterations, such as ret/PTC rearrangement or *B-RAF* mutation, may be necessary for the acquisition of the malignant phenotype, as in non-Hürthle cell papillary carcinomas (Grieco *et al*, 1990; Soares *et al*, 1998, 2003; Sugg *et al*, 1998; Cohen *et al*, 2003; Kimura *et al*, 2003).

The autosomal dominant inheritance of Hürthle cell tumours has been described in a single family (Canzian *et al*, 1998). A separate study of a large number of thyroidectomies for Hürthle cell tumours found that about 20% of cases showed multiple separate Hürthle cell tumours, and that these were significantly younger and much more often female than the solitary tumours (Katoh *et al*, 1998). No mutations were detected in any of the five exons, nor in the respective intronic boundaries of *GRIM-19* in the six cases from two Hürthle cell tumour families. Since four of these cases belonged to the family described by Canzian *et al* (1998), our results do not support the identification of *GRIM-19* as the *TCO* gene mapped to chromosome 19p13.2 by those authors (Canzian *et al*, 1998).

The observation that the only germline mutation in *GRIM-19* found in well over 100 samples tested was in the one patient with multiple Hürthle cell tumours suggests that this gene could account for some cases with a low penetrance pattern of inheritance. The absence of identifiable lesions in the patient's son who also carried the mutated gene is not surprising in view of his younger age (41 *vs* 60 years) and the rarity of multiple Hürthle cell tumours in males (Katoh *et al*, 1998).

Proof that a particular mutation is oncogenic requires both the demonstration of its presence in tumours, and the demonstration that the mutated gene confers an oncogenic advantage. We have shown that mutations in *GRIM-19* are associated with a specific subtype of thyroid tumours, and each of the four mutations we found led to amino-acid substitutions. Downregulation of *GRIM-19* has been shown to confer a growth advantage on cells and to reduce the likelihood that they will enter apoptosis (Angell *et al*, 2000). We therefore believe that *GRIM-19* mutations can make a significant contribution to the tumorigenic process in Hürthle cell tumours of the thyroid. Further *in vitro* studies are needed to demonstrate directly that the mutations we have found lead to downregulation of *GRIM-19* function.

## Figures and Tables

**Table 1:** Primer sequences used in GRIM-19 amplification and sequencing

	**Primer sequence (5′-3′)^a^**
*Exon-1*	GCA ACA CCC CAG AGG CAA GGT GA
AGA CTC TGA GAC CCC GGC GCA	

*Exon-2*	CAG TGT CCC CTG ATT GCA GAC
ACT TTC AGA CAA CGC CCA CCA	

*Exon-3*	GGT CTG ACC TGA GTG TGG GTT
CTT CCG GCC AGT GAC CTC CCA	

*Exon-4*	AGG CTT GAA GGG GTG CTA CTA
TCT GCC GTG GCT GGC ACC TCT	

*Exon-5*	GGT GGC TGT GCC TCT ACC CAT
AAA GGG GGT CAG GGG TCC TTT	

a For each exon, the first oligonucleotide represents the forward primer, and the second corresponds to the reverse primer.

**Table 2:** Summary of the data on the four cases with *GRIM-19* mutations

**Case**	**Age (years)**	**Diagnosis**	**Nucleotide alteration**	**Protein alteration**
4	42	HCFC	C77T	A26V
5	60	HCPC	G264C	K88N
6	33	HCPC	A247G	S83G
7	32	HCPC	G593C	R198P

HCFC=Hürthle cell variant of follicular carcinoma, HCPC=Hürthle cell variant of papillary carcinoma.

**Table 3:** Summary of the data on *ICAM1* relative expression in the four cases with *GRIM-19* mutations

**Case**	**Age (years)**	**Diagnosis**	***ICAM1* expression^a^ Tumour tissue *vs* normal tissue**
4	42	HCFC	3.2±0.4
5	60	HCPC	4.1±0.1
6	33	HCPC	3.3±0.3
7	32	HCPC	5.0±0.3

HCFC=Hürthle cell variant of follicular carcinoma, HCPC=Hürthle cell variant of papillary carcinoma.

a Values are expressed as the ratio between the expressions of *ICAM1* in tumoural tissues *vs* normal tissue, in mean±s.d. (after three measurements per case).

**Table 4:** Summary of the data regarding ret/PTC rearrangements and *B-RAF* mutations in the four patients with *GRIM-19* mutations

**Case**	**Age (years)**	**Diagnosis**	***B-RAF* mutation**	**RET/PTC rearrangements**
4	42	HCFC	Negative	Negative
5	60	HCPC	Negative	Negative
6	33	HCPC	Not done	Not done
7	32	HCPC	Negative	Positive

HCFC=Hürthle cell variant of follicular carcinoma, HCPC=Hürthle cell variant of papillary carcinoma.
